# Does Natural Amenity Matter on the Permanent Settlement Intention? Evidence from Elderly Migrants in Urban China

**DOI:** 10.3390/ijerph19031022

**Published:** 2022-01-18

**Authors:** Yanjiao Song, Nina Zhu

**Affiliations:** The Center for Modern Chinese City Studies, Institute of Urban Development, East China Normal University, Shanghai 200062, China; yjsong@iud.ecnu.edu.cn

**Keywords:** natural amenity, permanent settlement intention, elderly, migrants

## Abstract

This study focuses on the role of natural amenity in spurring the permanent settlement of elderly migrants in China, in the period from 2009 to 2017. Based on a combination of NASA’s Global Annual PM2.5 Grid data, and a nationwide China Migrants Dynamic Survey (CMDS) dataset, a binary logit model was used to investigate the settlement intention of migrants over 60 years old, across 291 cities in China. The empirical results revealed that there was a significant inverted U-shape between the annual temperature and permanent settlement, and prefectures with warmer winters and higher air quality were more attractive to elderly migrants when controlling for the urban endowment and economic conditions. In addition, the coefficient of the interaction term of air quality and precipitation was negative, indicating that the hindrance of precipitation on permanent settlement intention decreased with the enhancement in better air quality. Furthermore, there was significant group heterogeneity in the elderly’s migration reasons. The group of active movers cared more about environmental quality, whereas for the passive group, air quality had no effect on their permanent settlement.

## 1. Introduction

To date, elderly migration has become an important topic of concern to scholars worldwide [1,2,3]. Neoclassical economics theorizes that migration is the process of self-investment in human capital [4], although the motivations of older adults’ migration are more complicated. There has been a growing body of literature on the factors affecting elderly migration in the past decades [3,5,6]. According to the life cycle theory of elderly migration, the elderly, at around 60, migrate following retirement. Wiseman (1980) called this “amenity migration” in early retirement, which is usually from the northern states to the southern sunshine zone in America [7]. With the deterioration of older adults’ physical abilities, or the death of their spouse, “institutional” moves in late old age happen when elderly people move to live with close kin, or return to their original place of residence [2,3,8,9,10].

However, the patterns and motivations of elderly migration in China are quite different from retirement migration and institutional moves. One purpose of the elderly migration is to reunite with their children, which often involves helping look after their grandchildren and taking care of the housework [5,11,12]. According to the seventh national census, there are 260 million people aged 60 and over in China, accounting for 18.70% of the total population. The rapid growth of the scale of migrants has created the “old drifter”, which refers to an elderly migrant who moves from their rural homeland to a city, an important part of the urban new migrants. Therefore, studying the permanent settlement behavior of such a large-scale Chinese elderly population is crucial for understanding their needs for the future construction of urban public facilities.

In recent years, the number of elderly adults who care greatly about their quality of life has gradually increased [1,13,14]. The factors that affect the settlement choices of elderly migrants mainly include demographic characteristics, family-level factors, and urban benefits [7,8,9,11,12,15,16]. In addition, entertainment facilities and geographical distance also affect the migration of older adults [17]. As global climate change becomes a significant force, natural amenities have become an important indicator of human settlement choice [13,15]. Early research on the impact of climate change on human behavior has mainly focused on population migration caused by natural disasters and extreme climate events, such as earthquakes, tsunamis, hurricanes, flash floods, droughts, and fires [18,19,20]. Then, the concepts of “environmental migration”, “ecological migration”, and “climate migration” were further developed to strengthen the literature on the impact of climate and environmental changes on population migration [21,22,23,24,25].

The existing literatures show that temperature changes are closely related to migration. People are more likely to live in warm climates after retirement [26]. The migration rates of older adults in China were higher in the cold northern regions and lower in the warm southern areas [5]. Furthermore, seasonal mobility has been analyzed, with Chinese seasonal retirees motivated by natural amenities found to move to Sanya from the northeast cold region in winter [1]. For Swedish retirees, they spend their summers in Sweden and their winters in Spain [27]. Mueller et al. [28] studied population migration in eastern Africa and found that for every 1 standard unit increase in temperature deviation from the average temperature, the net emigration rate decreased by an average of 10 percentage points. Cai et al. [29] has suggested that in agriculture-based countries, rising temperatures will lead to a decline in the population migration ratio (population migration ratio means the ratio of the absolute amount of people moving in and out of a certain area in a certain period to the average population in that period and area). Some scholars verify that the impact of temperature changes on population migration is not a simple linear relationship, but rather a non-linear relationship. For example, Bohra-Mishra et al. [30] found that in Indonesia, there is a non-linear negative correlation between temperature changes and population migrations. When the temperature exceeds 25 °C, this increase in temperature will lead to a significant increase in population outflow.

Scholars have also conducted a lot of research on the impact of precipitation on migration. Similar to the impact of temperature changes on population migration, scholars also have different views on the impact of precipitation on migration. Some scholars believe that precipitation changes are positively correlated with population migration, whereas others hold the view that precipitation changes are negatively correlated with population migration [28,31,32]. Some scholars have proven that there is a U-shaped relationship between changes in precipitation and population migration [33], although there is no unified conclusion so far. Environmental quality also has an impact on population migration. Hunter [34] used data from 3109 counties in the United States from 1985 to 1990, and found that counties with higher environmental risks had lower migration rates. Zhang and Guldmann [35], using data from the Cincinnati metropolitan area in the United States from 1980 to 2000, found that air quality was a decisive factor in the choice of home location, and the higher the air quality in an area, the more attractive it was. Other results indicate that a 10-unit increase in PM2.5 concentration raises college graduates’ probability to leave their current city by 10% [6]. Xu [36] investigated the historical process of the impact of natural environment changes on population migration, suggesting that changes in the natural environment have an important impact on population migration, but in most cases, they are not a decisive factor. However, some scholars believe that the impact of the environment on population migration is not significant. For example, Cebula and Vedder [37] used data from 39 large metropolitan statistical areas in the United States from 1960 to 1968, and found that air pollution did not have a significant impact on population migration.

It can be seen that the movement choice of the elderly is motivated by the desires of a livable environment and elderly care services, but scholars do not share a unified conclusion on the impact of climate change on population migration, and the mechanism of how natural amenities affect elderly migration needs to be further studied. Besides, scholars mostly regard migration as a whole, and do not divide it into smaller units, such as dividing it into the migration activities of young and old people. Different types of populations respond differently to climate change. For example, young people possess a strong resistance to rising temperatures and increased pollution, whereas the elderly are more sensitive to these, as they can affect their health. It is necessary to discuss in detail the impact of climate change on different types of population migration. Moreover, the existing studies mainly focus on migration behavior, and the settlement choice of the elderly after migration needs to be further systematically studied.

Therefore, this paper aimed to examine the trend of the spatial distribution of elderly migrants and to verify the effects of natural amenity on their permanent settlement by using the national representative dataset of migrants in China. Conceptually, this paper found that rather than just having hedonic motivations, the migration motivations of the Chinese elderly are diversified. In addition to active migration, there is also passive migration due to family needs. This paper makes the first attempt to study elderly people’s passive migration and the permanent settlement intention in response to natural amenity in the Chinese context. Additionally, by focusing especially on natural amenity, this paper contributes to previous research that represents natural amenity as a set of multi-dimensional indicators, such as temperature, precipitation, and air quality, finding that there was an interaction effect of the explanatory variables on elderly migrants’ permanent settlement.

The remainder of the paper is organized as follows. Section 2 provides the data description, method, and the definition of variables. Section 3 reports the results of the empirical analysis, and Section 4 summarizes the main conclusions of this paper.

## 2. Data and Methodology

### 2.1. Data Description

The micro-statistical dataset of the Chinese elderly migrants used in this study came from the China Migrants Dynamic Survey (CMDS), which was released by the Migrant Population Service Center of the National Health Commission. This annual survey aimed at investigating the urban integration of the migrants who have currently resided in their immigratory city for more than one month, but have not registered in the district (county/city). To date, the CMDS is the most detailed microlevel survey data about Chinese elderly migrants. The questionnaire covered demographic characteristics, employment and social security, economic conditions and the settlement intention of migrants. The samples were obtained using the Probability Proportionate to Size Sampling (PPS) method with hierarchical, multi-stage, and scale proportions used, and they were representative, which provided a national data covering 31 provincial areas in China.

Based on the questionnaire, elderly migrants are defined as those who are over 60 years old, but are currently living in an inflow city. These respondents were required to answer questions such as “Are you willing to stay here for some time in the future?”, and “If you plan to stay here, how long do you plan to stay?”. In the original questionnaire, there were three types of answers for the former question: “Yes”, “No”, and “Not sure”. The six alternative answers for the latter question were “1–2 years”, “3–5 years”, “6–10 years”, “more than 10 years”, “living permanently”, and “not sure”. Respondents who decided to stay in the inflow city and were intent to live for more than five years were considered to have a permanent settlement intention, where the value of this variable was set to 1; otherwise, 0. Those migrants who “do not intend to stay in the inflow city”, were “not sure”, or “plan[ned] to live for less than 5 years”, were not considered to have a permanent settlement intention, and the value of this variable was set to 0.

The index of air quality was measured by the concentration of PM2.5 (in μg/m^3^), and the data mainly came from NASA’s Global Annual PM2.5 Grids data from 2009 to 2017, with the spatial resolution of 0.01° × 0.01°. We used ArcGIS 10.5 (Environmental Systems Research Institute, Inc., Redlands, CA, USA) and Python 3.6 (Python Software Foundation, Amsterdam, The Netherland) to match annual raster data to each city. The observed daily temperature (including daily maximum temperature and daily minimum temperature), and the daily precipitation data of 822 meteorological stations, were derived from the China Meteorological Data Service Center from 2009 to 2017 (http://data.cma.com, accessed on 21 August 2020). We used the Kriging interpolation method to interpolate the station data to the raster data, with a spatial resolution of 0.01° × 0.01°, and then ArcGIS and Python were used to match each year’s raster data to each city. Finally, based on the city index, this study matched the natural amenity data of each city with the microdata of the elderly migrants. The original samples including all migrants in 2015, 2016, and 2017 were 206,000, 169,000, and 169,989, respectively. After removing the default values and the invalid samples, there were 15,515 elderly migrants in the total sample, of which 6388 individuals were male and 9127 individuals were female.

### 2.2. Extreme Temperature Index

In order to describe the impact of temperature on the elderly’s willingness to settle in a more detailed manner, this paper not only analyzes the impact of the average temperature on the willingness of the elderly to settle, but also discusses the impact of extreme temperature on this willingness. There are different extreme temperature indices in the field of meteorology. Due to the limitation of the length of the article and the convenience of calculation, we selected two extreme temperature indices that are defined based on a threshold, namely the number of frost days (TN) and the number of summer days (TX) [38], and their definitions are as follows:

The number of frost days (FD): annual count of days when TN (daily minimum temperature) < 0 °C. Let TN*_ij_* be the daily minimum temperature on the day *i* in year *j*. Count the number of days where:

(1) $${\mathrm{TN}}_{ij}<0\;{}^{\circ}\text{C}$$

The number of summer days (SU): annual count of days when TX (daily maximum temperature) > 25 °C. Let TX*_ij_* be the daily maximum temperature on the day *i* in year *j*. Count the number of days where:

(2) $${\mathrm{TX}}_{ij}>25\;{}^{\circ}\text{C}$$

### 2.3. The Logit Model

We used the binary logistic regression model to conduct data analysis. The dependent variable was the urban settlement intention among elderly migrants, which was dichotomously coded as one if the elderly migrants were willing to settle in urban areas in the next five years, and zero if they were going back to their hometown or other cities. The specific model is expressed as follows:

(3) $$Y_{i}^{*}=\alpha_{i}+\beta_{i1}NATURE+\beta_{i1}X+\varepsilon_{i}\;\;$$

The subscript *i* in the logit model refers to the investigated individual in the local city. The dependent variable Y*_i_* in the model is measured by the elderly migrants’ intention to settle permanently in urban areas. In the study, we set the Y*_i_* to 1 if an elderly person chose to settle permanently in the city, whereas Y*_i_* was assigned to 0 if the elderly person chose not to settle, or had not decided yet. The independent variable in the model was the variable set NATURE. Previous studies have pointed out that population migration is affected by climate change and environmental pollution, and that these impacts will increase as people’s quality of life requirements increase [21,39,40]. To expand on these previous studies, this paper adopts multi-dimensional indicators to analyze the effect of natural amenity, which was represented by PM2.5 (μg/m^3^), number of summer days (SU) (day), number of frost days (FD) (day), annual temperature, and annual precipitation (mm).

The variable set X represented the control variables and was summarized into four levels: demographic variables, family-level factors, and natural amenities. The demographic variables mainly included age, age squared, gender, education level, the length of migration (year), and the distance of migration. For family characteristic variables, family income, costs, and the number of children was included in the logistic model. Furthermore, this paper has controlled city-level variables that would have affected the elderly’s settlement intention, such as the establishment of health records in local cities, housing prices, and the economic development of a given city as defined by gross domestic product (GDP). Considering that the migration of the elderly may be closely linked to rapidly deteriorating health conditions, which often begin with hospitalization [2], the number of beds in the city was also controlled. The variable definitions and descriptive statistics are reported in Table 1.

Based on Table 1, we further provide the statistical analysis of the differences in elderly migrants with and without permanent settlement intention. Among the elderly participants aged above 60, 67.43% of the elderly migrants reported that they were planning to stay permanently in the city they had chosen. On average, elderly women were more willing to settle in cities than men, with the probabilities for the former and latter being 68.67% and 66.58%, respectively. According to the completed years of formal education, the elderly migrants were divided into four categories: primary school and below, junior middle school, senior high school, and university and above. The possibilities of settlement intention for the four groups were 63.92%, 68.44%, 72.25%, and 80.46%, respectively. The elderly’s permanent settlement likelihood was also related to the nature of household registration, migration duration, and migration distance, and so on. The results from the CMDS showed that, with non-agricultural household registration, the longer the migration duration and the shorter the migration distance, the higher the willingness of the elderly to settle permanently in the city. The establishment of urban health archives was also found to be conducive to promoting the settlement of elderly migrants. The urban settlement intention of the group with archives was 5.73 percentage points higher than that of the group without health archives. However, the proportion of cities which had established health archives for elderly migrants, at only 43.88%, was obviously insufficient.

## 3. Empirical Analysis

### 3.1. The Spatiotemporal Changes of Elderly Migrants and Natural Amenity

Table 2 shows the time trend of settlement intention and natural amenity during the period of 2015–2017. From a national perspective, it can be seen that the settlement intentions of the elderly were 0.68, 0.73, and 0.62 in 2015, 2016, and 2017, respectively. Furthermore, the mean value of the settlement intention of the elderly was 0.67, indicating that most elderly people who migrated to other cities were more willing to stay in the local areas chosen. PM2.5 also showed a decreasing trend on the whole, especially after 2014, mainly because China promulgated the “Air Pollution Prevention and Control Action Plan” in 2013, when government departments and enterprises took various actions to prevent and control regional pollution. It can be seen that a series of policies to reduce PM2.5 adopted in recent years have achieved good results in China. The mean value of PM2.5 was 42.401 μg/m^3^, which is lower than the excellent standard of 50 μg/m^3^, indicating that China’s environmental quality during this period was at a good level, on the whole.

In the context of global warming, SU and precipitation showed an increasing trend: the change slopes were 0.178 and 18.007, respectively, whereas FD showed a decreasing trend in China from 2009 to 2017: its change slope was −0.589. The values of SU fluctuated between 41 and 50. Among them, SU in 2013 was the largest, and about 14% of the days in a year belonged to extremely high temperature conditions. The values of FD fluctuated between 31 and 40, and about 11% of the days in a year belonged to extremely low temperature conditions. There were ten more summer days than frost days on the whole, and the gap between the two will become larger and larger in the context of global warming. In addition, the mean value of precipitation was 977.009 mm, precipitation in 2011 was the lowest with a value of 823.257 mm, and precipitation in 2016 was the largest with a value of 1139.313 mm, indicating that the precipitation values did not change significantly from 2009 to 2017 (Note: The survey period for natural amenity used in this article started in 2009, which is earlier than the micro data survey of the elderly migrants. There are two main reasons for this: first, the earliest survey data of the CMDS was in 2009, but the maximum age of the surveyed individuals in this database was 60 years old, so this article started from the available data in 2015. Second, there was a certain lag in the effect of air pollution control, and the time effect of natural amenity can be seen more clearly by adding the data of the previous years).

**Figure 1:**
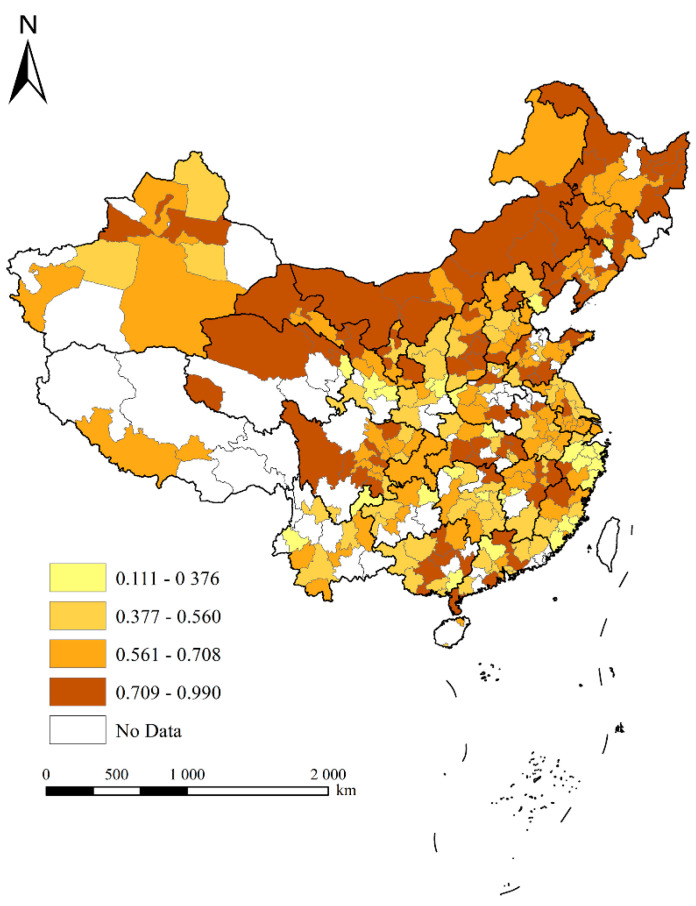
The spatial pattern of the settlement intention of the elderly people.

**Figure 2:**
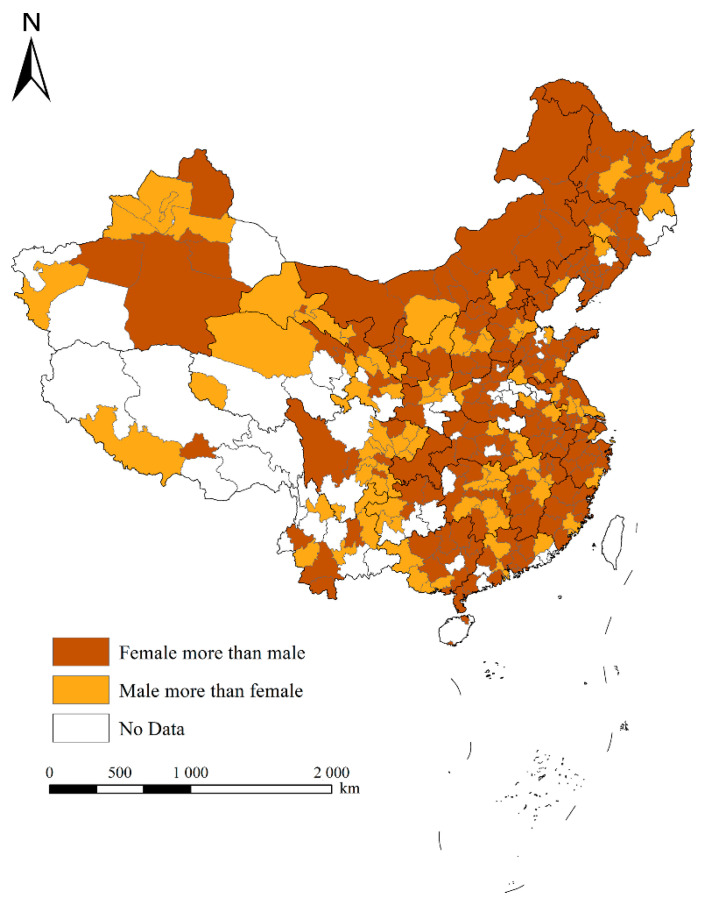
The spatial pattern of the settlement intention of the gender ratio of elderly people.

In space, Figure 1 and Figure 2 represent the spatial pattern of the settlement intention of the elderly and its gender ratio, respectively. Figure 1 shows that the high-value areas of the settlement intention of the elderly were mainly distributed in the northern region of China, such as Heilongjiang, Jilin, Liaoning, Beijing, Inner Mongolia, etc., as well as some scattered cities in the central and western regions, such as Sichuan, indicating that the elderly mainly wanted to live in these areas after retirement. The low-value areas of the settlement intention of the elderly were mainly distributed in some cities along the southeast coast, such as Ningbo, Taizhou, and Wenzhou, and some scattered cities in the central and western regions, indicating that these cities were not popular with the elderly. It can be seen from Figure 1 that although the economic development level, medical level, and infrastructure conditions in eastern China were found to be relatively good for the elderly, they did not pay much attention to those aspects, but preferred to choose to live in some places with better natural amenity, which is different from the choices of young people [40]. In addition, for most cities, the average ratio of women’s willingness to settle was higher than that of men, indicating that women were more likely to move to non-native places after retirement. Among them, the proportion of women’s willingness to settle in some cities in the eastern region was significantly higher than that of men, whereas the proportions of women’s and men’s willingness to settle in cities in the central and western regions were similar. It is worth noting that the proportion of men’s willingness to settle was higher than that of women in Xinjiang.

**Figure 3:**
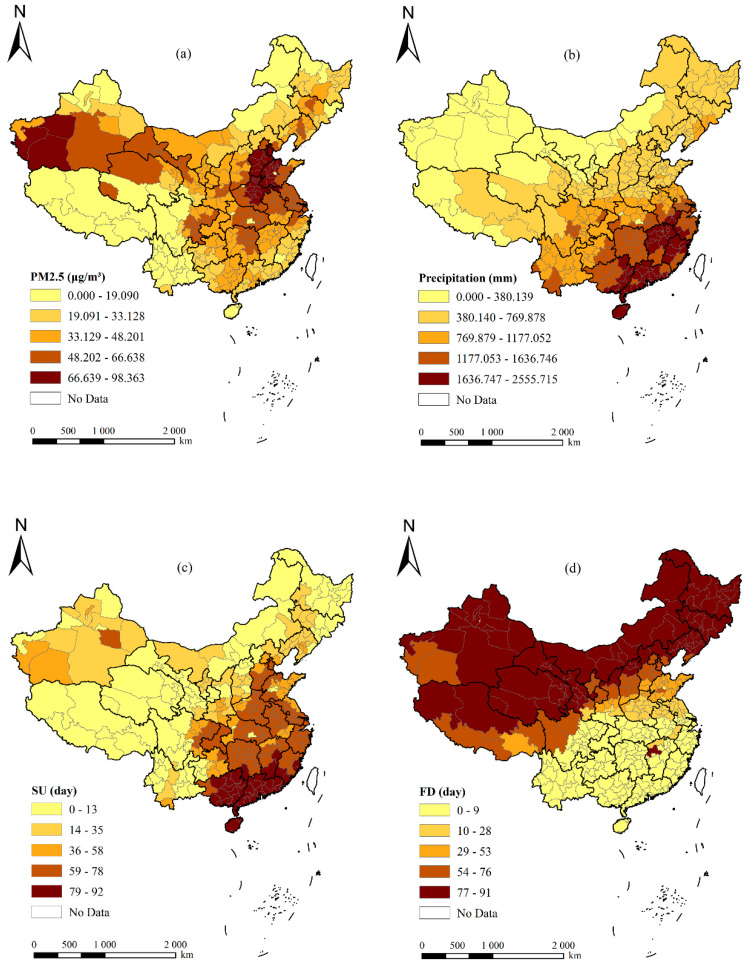
The spatial patterns of PM2.5 (μg/m^3^) (**a**), precipitation (mm) (**b**), number of frost days (day) (**c**), and number of summer days (day) from 2009 to 2017 (**d**).

Furthermore, Figure 3 also provides the spatial patterns of PM2.5 (μg/m^3^) (a), precipitation (mm) (b), SUs (day) (c), and FDs (day) (d) from 2009 to 2017. From Figure 3a, we can see that there were two high-value areas for PM2.5, which were distributed in the Bohai Bay and Xinjiang, respectively, and that PM2.5 values decreased toward the periphery, with these two high-value areas being the center. The low-value areas for PM2.5 were mainly distributed in Northeast China and Southwest China. There was relatively little heavy industry pollution in these areas, the vegetation coverage was high, and the ecological environment was good. Therefore, the concentration of PM2.5 was low. The spatial pattern of precipitation was similar to that of the SUs; both showed a gradually decreasing trend from southeast to northwest (Figure 3b,c). Due to the special physical conditions of the elderly, hot and humid weather can easily induce rheumatism, gastrointestinal diseases, respiratory diseases, skin diseases, and cardiovascular diseases. Therefore, the southeast coastal areas in China are generally not suitable migration locations for the elderly. On the contrary, the spatial pattern of FDs showed a gradually increasing trend from southeast to northwest. However, FDs gradually decreased because of global warming, according to Table 1, and the impact of extremely low temperatures on the elderly is expected to become smaller and smaller.

### 3.2. The Empirical Results Based on the Logit Model

#### 3.2.1. Natural Amenities and Elderly Migrants’ Permanent Settlement

Table 3 reports the coefficients of the marginal effect and robust standard errors of natural amenity on the permanent settlement intention of elderly migrants. By using the binary logit model, this study adopted the step-by-step regression method and analyzed the impact of natural amenity on the permanent settlement intention of elderly migrants by gradually controlling factors such as individual characteristics, family factors, and natural amenity in Model 1–5. Compared with elderly migrants who were not willing to permanently settle in the local cities (hereafter referred to as the “unwilling” group), the results in Model 1 indicate that precipitation had a significant negative impact on the probability of elderly people being willing to settle in a city (the “willing” group), that is, cities with less rainfall were found to be more attractive to the elderly. The relationship between the annual average temperature and the elderly’s willingness to settle down was also found to be negative at the significant level of 1%. In view of certain scholars who believe that the relationship between temperature and migration is not linear [30], the square term of annual average temperature was added in Model 2. The results suggest that there was a significant inverted U-shape between the annual average temperature and the settlement activity of the elderly migrants. The air quality was measured by PM2.5, which refers to particles with an aerodynamic equivalent diameter of less than 2.5 microns in the environment. The higher the value of PM2.5 in the air, the more serious the air pollution. After considering the control variables at the individual, family, and urban levels, cities with worse air quality were found to be less attractive to the elderly.

The results of the control variables show that there was a significant inverted U-shape between age and the settlement willingness of the elderly migrants. According to the calculation, the optimal age, when the elderly population had the highest willingness to settle, was 73.5 years old. After that, the willingness to settle down showed a downward trend. A possible explanation for this is that when an elderly person reaches a certain age, their physical functions decline, and they may return to the familiar environment of their hometown for elderly care, or to seek help from their children. In addition, those with a high willingness to settle were generally women with higher levels of education and non-agricultural households. Previous research has verified that elders with a greater number of adult children were more likely to relocate [17]. Therefore, this paper controlled the family size (measured by the number of children) in Model 4. The results show that the number of children in the family was conducive to the permanent settlement of the elderly migrants in local cities. In addition, urban medical services would also affect the willingness of the elderly to settle. Model 5 further added the number of beds per thousand people in the city, and the estimated marginal effects were positive, which indicates that the more convenient the urban medical services were, the more likely it was for the elderly to choose to settle down.

#### 3.2.2. Extreme Climate Conditions and Elderly Migrants’ Permanent Settlement

In Table 4, the first and fourth columns report estimates in which the annual average temperature was replaced by extremely hot days (temperatures above 25 °C in summer) and extremely cold days (temperatures below 0 °C in winter) to better understand the elderly’s response to extreme climate conditions in different seasons. The days with temperatures lower than 0 °C in winter were defined as frost weather, and the days with temperatures higher than 25 °C in summer were regarded as summer weather. According to the CMDS database, there are 34 cities that have recorded the highest temperature below 25 °C in summer, and 139 cities that have recorded a highest temperature above 0 °C in winter in China. The results in Model 6 and Model 7 show that extreme hot weather had no significant effect on the settlement intention of the elderly. However, extreme frost weather in winter was found to significantly hinder the elderly from settling in the city. Model 8 describes the impact of excellent air quality on the behavior of the elderly migrants. The cities with PM2.5 values lower than 50 were defined as being in the excellent weather category, whereas the cities with PM2.5 values greater than 50 were regarded as being in the reference group. The results of Model 8 indicate that excellent air quality was conducive to the settlement of the elderly, which further validates the conclusions in Table 2 above.

### 3.3. Analysis of the Mechanisms

From the estimated results shown in Table 3, it can be seen that the precipitation, average annual temperature and air quality all had a significant impact on the settlement intentions of the elderly. However, based on the existing research on air pollution, air quality is affected by the interaction of natural, economic, and social conditions. Air pollutants mainly come from emissions generated by economic and social development, and are affected by natural factors such as rainfall and seasonal temperature. In order to control the interaction between explanatory variables and the potential endogeneity of air pollution and urban medical services, the interaction terms of air quality and precipitation, air quality and frost weather, and air quality and the number of hospital beds, were used as moderator variables. In view of the comparative analysis of coefficients of the independent variables before and after adding the interaction terms, this paper adopted the method of Balli et al. [41], and each observation was de-averaged. The conclusions show that the marginal effect coefficient of the interaction term of air quality and precipitation was significantly negative, indicating that the hindrance of precipitation on permanent settlement intention decreased with the enhancement of excellent air quality. In addition, this paper found that with the addition of the interaction term of air quality and precipitation, the inverted U-shaped trend of temperature on settlement intention was still significant, but the effect of precipitation on the settlement decisions of the elderly became insignificant (see Model 10 and Model 11 in Table 5). The marginal effect coefficients of air quality and temperature were not significant, which means that the attraction of a city with pleasant air quality to the elderly was not affected by the frost days in winter.

### 3.4. The Dynamic Results of Natural Amenity and the Migration of the Elderly

From the above empirical estimates, we can observe that the indicator of air quality played a key role in influencing the urban settlement willingness of the elderly. The “China Smog” has attracted the attention of all public view sectors since around 2010. Haze has a certain harm to the economy and human behavior, especially the health of the elderly. In September 2013, the State Council issued the action plan for the prevention and control of air pollution. With the regulation of air pollution by the Chinese government, the annual average values of PM2.5 decreased from 45.12 in 2014 to 37.58 in 2016, suggesting that urban air quality is gradually improving in China (see more details in Table 2). Therefore, in Model 12–Model 14, the interaction term of air quality and urban GDP was introduced to control the regulatory effect of urban economy on air pollution, and the effect of dynamic changes of natural amenity on the behavior of the elderly was analyzed by three sub-samples from 2015 to 2017.

The results in Table 6 show that air quality did not have a significant impact on the relocation of the elderly in 2015. However, in the following years, the elderly began to pay attention to urban air quality when choosing urban settlement. More interestingly, the impact of the number of hospital beds on the settlement of the elderly has also become significant since 2016, although the number of hospital beds had no impact on the relocation of the elderly in previous years. This reveals that the Chinese elderly have begun to realize the improvement of their own welfare in recent years, focusing on welfare factors such as health-related natural endowment factors and urban public services. The results of the coefficient of health archives in Columns 1, 2, and 3 further verify this viewpoint.

### 3.5. Heterogeneity Analysis by Flow Reason

The elderly in China are a large group, with complex migration motives. It is important to consider the heterogeneity of the elderly, and examine whether natural amenities affect different groups of elderly people differently. According to the questions about the reasons for elderly migration in the questionnaire, the migration motives included working, business, moving with families, taking care of the elderly or children, and retirement migration. Based on the subjectivity of migration motivation, groups such as migrant workers, engaging in business or retirement for old-age care in other places, were defined as active migrants. Those elderly groups who migrated with families, to take care of family members and so on, were defined as passive migrants. Table 7 compares results from the two sub-sample models, revealing a contrasting story regarding the results for the location choices of the elderly for active migrants and passive migrants. The heterogeneity analyses of the flow reasons of elderly migrants had two interesting findings: first, active migrants were found to pay more attention to environmental quality. For example, the number of days with excellent air quality every year was found to help this group to settle permanently in the city, but this factor was not significant for passive migration groups. For the active migrants, urban medical convenience was apparently an important determinant of retirement migration, whereas the number of beds in urban hospitals had no significant effect on the passive migrants’ permanent settlement decisions. This suggests that, when making permanent settlement choices, active migrants pay more attention to the needs of their own welfare, whereas the latter care more about the needs of family members.

## 4. Conclusions

This study investigated the effect of natural amenity on elderly migrants’ permanent settlement by using data from the CMDS. We adopted multi-dimensional indicators to measure the natural amenities, which were represented by PM2.5, the number of summer days, and annual precipitation (mm). Based on the binary logit model and a robustness check, the empirical results revealed that natural amenities play an important role in the permanent settlement of elderly migrants in urban China. The main findings of this paper are summarized as follows.

First, over time, SUs and precipitation showed an increasing trend, while the settlement intention of the elderly and FDs showed a decreasing trend in China from 2009 to 2017. Spatially, the high-value areas favored by the elderly as migration locations were mainly distributed in the northern region of China, as well as some scattered cities in the central and western regions. The low-value areas were mainly distributed in some cities along the southeast coast.

Second, there was a significant inverted U-shape between the annual average temperature and the settlement decisions of the elderly migrants, and cities with worse air quality were less attractive to the elderly. Specifically, prefectures with warmer winters and better air quality were more attractive to elderly migrants when controlling for the urban endowment and economic conditions.

Third, among the variables of natural amenity, air quality and precipitation had a regulatory effect on elderly permanent settlement. The empirical results show that the marginal effect coefficient of the interaction term of air quality and precipitation was significantly negative, indicating that the hindrance of precipitation on permanent settlement intention decreased with the enhancement of excellent air quality.

Fourth, there was significant group heterogeneity in the impact of natural amenity on permanent settlement. The elderly migrants who moved actively cared more about environmental quality, whereas for the passive group—who moved to help their children take care of grandchildren—air quality had no effect on their permanent settlement decisions. This indicates that when making permanent settlement choices, active migrants paid more attention to the needs of their own welfare, whereas the latter cared more about the needs of family members.

With the promulgation of the universal three-child policy in China, the family demand for childcare from grandparents will increase significantly, and the scale of elderly migration groups will continue to grow. From the perspective of migration motivations, the hedonic migration of the elderly in China is not the primary motivation, which is very different from the findings of previous analyses regarding the mechanisms of retirement migration in the United States and other countries. Moreover, although the number of elderly migrants is huge, they cannot enjoy the corresponding urban elderly care services, because of China’s unique *Hukou* system in megacities. In China, the urban public services are closely related to the local registered permanent residence system. That is to say, even though migrants have lived in cities for many years and have paid urban social insurance, they cannot enjoy the corresponding social security services if they do not obtain the urban hukou of the city where they live. In the future, local governments can continuously improve their elderly care systems in combination with natural endowments, which are both factors valued by the elderly. The medical public services for elderly migrants need further innovation, and the inter-provincial payment system for medical security services should also been fully opened, so as to enhance the convenience of elderly migrants’ permanent settlement in cities across China.

## Figures and Tables

**Table 1:** The descriptive statistics of variables.

	Variables	Definition of Variables	Sample Size ^1^	Mean	SD	Min	Max
Dependent variable	Settlement intention	The willingness to settle in urban areas in the next five years (0 = No; 1 = Yes)	15,515	0.67	0.47	0	1
Independent variables	Air quality	Annual PM2.5	1023	43.39	15.37	3.46	91.84
	Temperature	Annual temperature	1023	12.90	5.42	−2.90	25.30
	Extreme low temperature	Number of frost days per year	1023	41.80	27.34	0	92
	Extreme high temperature	Number of summer days per year	1023	43.66	39.01	0	91
	Precipitation	Annual precipitation (mm)	1023	891.11	533.47	68.45	3053.84
Control variables	Age	Above 60 years old	15,515	65.83	5.58	60	98
	Age	Age squared	15,515	4365.39	776.99	3600	9604
	Gender	0 = male; 1 = female	15,515	0.41	0.49	0	1
	Education	Completed years of formal education in regular school	15,515	7.46	4.22	0	19
	*Hukou* ^2^	0 = non-agricultural Hukou; 1 = agricultural Hukou	15,513	0.58	0.49	0	1
	Expenditure	Monthly household consumption	15,513	2991.92	3187.32	50	10,500
	Income	Monthly household income	15,515	5685.83	10,241.05	−1000	100,000
	Health archives	Whether a health record was established in the local city (0 = No; 1 = Yes)	14,514	0.44	0.50	0	1
	Family size	The total number of biological children	15,515	2.71	1.37	1	10
	Length of migration	Years of migration	15,515	8.08	7.75	0	81
	Distance of migration ^3^	1 = intra-provincial migration; 2 = inner-provincial migration; 3 = inner-city migration	15,509	1.80	0.78	1	3
	Housing price	Average commercial housing price of the city	1023	9848.54	8248.17	2245.45	47,936
	GDP	Gross domestic product (100 million Yuan)	1023	8049.06	9172.54	34.95	30,632.99
	Hospital facilities	The number of beds in medical and health institutions (per thousand)	1023	37.92	41.65	0.12	142.71

Note: ^1^ The total sample size of the city-level variables (including independent variables, housing price, GDP, hospital facilities) is calculated as follows: N = n × t, where n refers to the number of 341 cities and t refers to the year (2015, 2016, 2017). ^2^ *Hukou* refers to the registered permanent residence system in China. ^3^ We calculated the sample weight of the distance of migration based on three types: intra-provincial migration, inner-provincial migration, and inner-city migration. The proportion of inter-provincial migration is the highest at 42.50%; the second is intra-provincial migration of 34.82%. The proportion of intra-city migration is only 22.68%.

**Table 2:** Changes in elders’ settlement intention and natural amenity during the period of 2009–2017.

Year	Settlement Intention	PM2.5 (μg/m^3^)	SU (Day)	FD (Day)	Precipitation (mm)
2009	-	43.738	46.000	34.000	875.684
2010	-	44.381	45.000	35.000	1033.332
2011	-	41.520	45.000	40.000	823.257
2012	-	39.336	45.000	40.000	1008.093
2013	-	44.952	49.000	36.000	951.092
2014	-	45.116	41.000	34.000	958.653
2015	0.680	41.677	42.000	32.000	1026.735
2016	0.730	37.580	49.000	34.000	1139.313
2017	0.620	43.313	49.000	31.000	976.922
Mean	0.670	42.401	46.000	35.000	977.009
Slope	−0.03	−0.267	0.178	−0.589	18.007

Note: Table 2 is city-level data; SU means the number of summer day and FD means the number of summer days.

**Table 3:** The effect of natural amenity on elderly migrants’ urban settlement.

Variables	Model 1	Model 2	Model 3	Model 4	Model 5
	Margin Effects	Margin Effects	Margin Effects	Margin Effects	Margin Effects
Precipitation	−0.135 ***	−0.146 ***	−0.148 ***	−0.112 **	−0.147 **
	(−3.33)	(−3.45)	(−3.46)	(−2.41)	(−2.44)
Temperature	−0.026 ***	0.008	0.016	0.022	0.039 *
	(−4.99)	(0.52)	(1.01)	(1.26)	(1.77)
Temperature squared		−0.001 **	−0.002 **	−0.002 **	−0.002 **
		(−2.05)	(−2.45)	(−2.20)	(−2.50)
PM2.5	0.002	0.001	−0.001	0.003 *	−0.004 **
	(1.58)	(0.80)	(−0.62)	(1.70)	(−2.04)
Age			0.182 ***	0.165 ***	0.147 **
			(3.12)	(2.64)	(2.04)
Age squared			−0.001 ***	−0.001 **	−0.001 *
			(−2.66)	(−2.27)	(−1.71)
Gender (base group: male)			0.109 ***	0.139 ***	0.162 ***
			(3.00)	(3.54)	(3.70)
Junior middle school (base group: primary and below)			0.126 ***	0.204 ***	0.189 ***
			(2.94)	(4.34)	(3.63)
High school (base group: primary and below)			0.154***	0.248***	0.311***
			(2.63)	(3.89)	(4.41)
College and above (base group: primary and below)			0.482 ***	0.642 ***	0.677 ***
			(4.91)	(6.12)	(5.97)
Agricultural *Hukou*			−0.522 ***	−0.570 ***	−0.554 ***
			(−12.45)	(−12.07)	(−10.58)
Ln (expenditure)			0.061	0.366 ***	0.353 ***
			(1.34)	(8.72)	(7.57)
Ln (income)			−0.135 ***	−0.282 ***	−0.258 ***
			(−3.16)	(−7.18)	(−5.78)
Health archives				0.195 ***	0.186 ***
				(4.94)	(4.19)
Family size				0.057 ***	0.058 ***
				(3.40)	(3.09)
Length of migration				0.074***	0.077 ***
				(23.12)	(21.16)
Inner-provincial migration (base group: intra-provincial migration)				0.301 ***	0.275 ***
				(6.82)	(5.13)
Inner-city migration (base group: intra-provincial migration)				0.413 ***	0.433 ***
				(7.99)	(6.98)
Ln (Housing prices)				−0.034	−0.060
				(−0.66)	(−0.94)
Ln (GDP)				−0.301 ***	0.040
				(−6.09)	(0.82)
Ln (Beds)					0.089 *
					(1.94)
2016 (base group: 2015)		0.109 **			−0.087
		(2.44)			(−1.54)
2017 (base group: 2015)		−0.088 **			−0.328 ***
		(−2.10)			(−5.32)
_cons	1.870 ***	1.788 ***	−5.040 **	−6.192 ***	−6.288 **
	(7.78)	(6.67)	(−2.48)	(−2.80)	(−2.47)
*N*	15,515	15,515	15,513	14,337	11,734

Note: ***, **, and * indicate significance at the levels of 1%, 5%, 10%, respectively. The abbreviation “Ln” is the logarithmic form.

**Table 4:** The effect of extreme climate conditions on elderly migrants’ settlement intention.

Variables	Model 6	Model 7	Model 8
	Extreme Hot Weather	Extreme Cold Weather	Excellent Air Quality
Precipitation	−0.151 **	−0.169 ***	−0.148 **
	(−2.50)	(−2.78)	(−2.46)
Temperature	0.037 *	0.040 *	0.030
	(1.69)	(1.82)	(1.48)
Temperature squared	−0.002 **	−0.003 ***	−0.002 **
	(−2.44)	(−2.89)	(−2.29)
Summer weather	0.012		
	(0.10)		
Frost weather		−0.163 **	
		(−2.17)	
PM2.5	−0.004 **	−0.003	
	(−1.98)	(−1.53)	
Excellent air quality			0.120 **
			(2.23)
Control variables	YES	YES	YES
_cons	−6.202 **	−5.923 **	−6.599 ***
	(−2.43)	(−2.33)	(−2.58)
*N*	11,734	11,734	11,734

Note: ***, **, and * indicate significance at the levels of 1%, 5%, 10%, respectively.

**Table 5:** The interaction effects of natural amenity on elderly migrants’ settlement intention.

Variables	Model 9	Model 10	Model 11
	Margin Effects	Margin Effects	Margin Effects
Air quality × Precipitation		−0.279 ***	−0.250 ***
		(−4.57)	(−3.84)
Air quality × Frost weather			0.164
			(1.51)
Air quality × Beds			0.020
			(0.45)
Precipitation	−0.170 ***	−0.011	−0.011
	(−2.78)	(−0.54)	(−0.53)
Temperature	0.035 *	0.029	0.029
	(1.71)	(1.45)	(1.42)
Temperature squared	−0.002 ***	−0.002 **	−0.002 **
	(−2.87)	(−2.24)	(−2.19)
Frost weather	−0.174 **	−0.132 *	−0.218 **
	(−2.32)	(−1.76)	(−2.35)
Excellent air quality	0.111 **	1.975 ***	1.459 **
	(2.05)	(4.80)	(2.36)
Health archives	0.183 ***	0.178 ***	0.177 ***
	(4.12)	(4.00)	(3.97)
Ln (Bed)	0.066	0.092 **	0.081
	(1.43)	(1.97)	(1.39)
Control variables	YES	YES	YES
_cons	−6.715 ***	−7.730 ***	−7.405 ***
	(−2.63)	(−3.05)	(−2.92)
*N*	11,734	11,734	11,734

Note: ***, **, and * indicate significance at the levels of 1%, 5%, and 10%, respectively.

**Table 6:** The dynamic results of natural amenity and elderly migrants’ settlement intention.

Variables	Model 12	Model 13	Model 14
	2015	2016	2017
Air quality × GDP	0.223 **	−0.097	0.031
	(2.23)	(−1.09)	(0.54)
Air quality × Precipitation	−0.583 ***	−0.483 **	−0.410 ***
	(−3.79)	(−2.46)	(−2.83)
Excellent air quality	0.044	5.250 ***	2.282 *
	(0.03)	(2.68)	(1.92)
Frost weather	0.008 **	−0.000	0.002
	(2.07)	(−0.12)	(0.74)
Precipitation	0.426 **	0.274	0.244
	(2.16)	(1.35)	(1.41)
Temperature	0.049	0.044	0.139 ***
	(0.91)	(1.01)	(3.72)
Temperature squared	−0.002	−0.002	−0.00 ***
	(−0.98)	(−1.36)	(−3.99)
Health archives	−0.403 ***	0.681 ***	0.210 ***
	(−4.52)	(8.31)	(2.73)
Ln (Bed)	0.064	0.174 **	0.196 *
	(0.72)	(2.26)	(1.94)
Control variables	YES	YES	YES
_cons	−0.335	−13.96 ***	−12.61 ***
	(−0.06)	(−2.88)	(−3.06)
*N*	3347	4051	4336

Note: ***, **, and * indicate significance at the levels of 1%, 5% and 10%, respectively.

**Table 7:** The natural amenity and elderly migrants’ settlement by migration reason.

Variables	Model 15	Model 16
	Active Migrants	Passive Migrants
Precipitation	0.149	0.262
	(1.04)	(1.54)
Temperature	−0.067 *	0.104 ***
	(−1.81)	(2.76)
Temperature squared	0.002	−0.004 ***
	(1.33)	(−3.00)
Frost weather	−0.001	0.004
	(−0.49)	(1.45)
Excellent air quality	1.966 *	0.979
	(1.75)	(0.74)
Air quality × Precipitation	−0.517 ***	−0.334 **
	(−4.21)	(−2.21)
Air quality × Frost weather	0.051	0.024
	(1.23)	(0.51)
Air quality × Beds	0.076	0.078
	(1.30)	(1.15)
Health archives	0.135 **	0.223 ***
	(2.15)	(3.17)
Ln (Bed)	0.146 **	0.005
	(2.28)	(0.06)
_cons	−6.864 *	−4.315
	(−1.72)	(−1.09)
*N*	5589	5392

Note: ***, **, and * indicate significance at the levels of 1%, 5%, 10%, respectively.

## Data Availability

The original data of this study was obtained from the Migrant Population Service Center, National Health Commission China. We are authorized to use the data by submitting a formal application to the Migrant Population service Center in December 2020, and the data was available online at the website http://www.chinaldrk.org.cn (accessed on 24 November 2020).
